# Fingerprint image enhancement using multiple filters

**DOI:** 10.7717/peerj-cs.1183

**Published:** 2023-01-03

**Authors:** Haroon Shams, Tariqullah Jan, Amjad Ali Khalil, Naveed Ahmad, Abid Munir, Ruhul Amin Khalil

**Affiliations:** 1 Department of Electrical Engineering, University of Engineering & Technology Peshawar, Peshawar, Pakistan; 2 College of Computer and Information Sciences, Prince Sultan University, Riyadh, Saudi Arabia; 3 Department of Electronic Engineering, The Islamia University of Bahawalpur, Bahawalpur, Pakistan

**Keywords:** Biometric, Coherence diffusion filter, Fingerprint, Image enhancement, Gabor filter, Log-Gabor filter

## Abstract

Biometrics is the measurement of an individual’s distinctive physical and behavioral characteristics. In comparison to traditional token-based or knowledge-based forms of identification, biometrics such as fingerprints, are more reliable. Fingerprint images recorded digitally can be affected by scanner noise, incorrect finger pressure, condition of the finger’s skin (wet, dry, or abraded), or physical material it is scanned from. Image enhancement algorithms applied to fingerprint images remove noise elements while retaining relevant structures (ridges, valleys) and help in the detection of fingerprint features (minutiae). Amongst the most common image enhancement filters is the Gabor filter, however, given their restricted maximum bandwidth as well as limited range of spectral information, it falls short. We put forward a novel method of fingerprint image enhancement using a combination of a diffusion-coherence filter and a 2D log-Gabor filter. The log-Gabor overcomes the limitations of the Gabor filter while Coherence Diffusion mitigates noise elements within fingerprint images. Implementation is done on the FVC image database and assessed *via* visual comparison with coherence diffusion used disjointedly and with the Gabor filter.

## Introduction

Identity is an important aspect of everyday life; hence individuals are assigned a classification for identification. A surname identifies the family, while a first name distinguishes one from the immediate family members to which an individual belongs. Other identifiers such as age, height, skin tone, and ethnicity improve reliability in identification but an identifier that is linked to the biological makeup of an individual, *i.e.,* biometrics, will have higher reliability as it is not easy to tamper with. The ability to correctly identify or authenticate an individual based on the physical physiology or behavioural makeup has a higher degree of accuracy when compared to knowledge-based or token-based *i.e.,* passwords or keys. Biometrics can be stated as the measurement and analysis of unique physical or behavioural characteristics like fingerprint or voice patterns, especially as means of verifying personal identity (Merriam-Webster, 2022; Bhatti et al., 2022; Khalil et al., 2015). Some of the most common types of biometrics are auditory biometrics, behavioural biometrics, chemical biometrics, and visual biometrics. In auditory biometrics the voice is used to determine the identity of the speaker, while in behavioural biometrics one can identify an individual on their gait *i.e.,* walking style. Chemical biometrics includes DNA matching, by analyzing a segment of DNA an individual can be identified. Visual biometrics identify individuals by Iris recognition using the eye’s iris features for identification, retina recognition using patterns of veins behind the eye for identification, and fingerprint recognition using the patterns on the fingerprint as a form of identification (Jain, Ross & Prabhakar, 2004). One of the important categories of visual biometrics is fingerprint identification employed in a number of commercial applications. There are several biometric identifiers namely DNA, face recognition, fingerprints recognition, iris recognition, and retina recognition, but preference and important factors are given to those biometrics with ease of access as well as reliability. This is where fingerprints stand out, as the probability of two individuals with the same fingerprint is extremely low, while at the same time fingerprints have ease of access. The need for security in personal identification increases with the advent of newer forms of communication in applications such as banking systems, e-commerce, mobile phones, smart cards, *etc*. Amongst biometric traits, identification *via* fingerprints has the gold standard of reliability and combined with the ease of capturing, storing, and, matching fingerprint (Jain & Pankanti, 2001).

### Fingerprint

**Figure 1:**
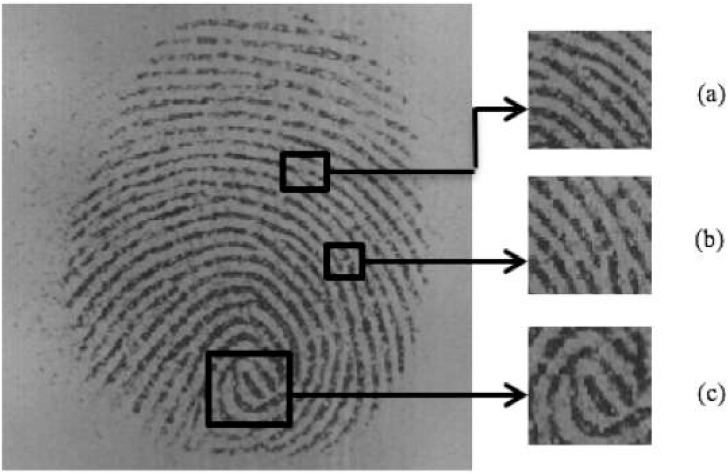
Fingerprint image with highlighted minutiae (A) ridge bifurcation (B) ridge ending (C) singular region.

The skin covering the surface of a human hand and foot is patterned with ridges which are curved protruding single segments. Two parallel ridges are separated by a gap called a valley. Ridges and valleys form curved, complicated, and unique patterns that help in the exudation of perspiration, allow for gripping as well as allow for a sense of touch (Datta et al., 2001; Noor et al., 2018). Ridges have small pores that extrude perspiration, when an item is picked or touched, the perspiration leaves an exact impression *i.e.,* latent print, of the ridges on the object. The latent print depends on the object’s surface as well as environmental factors such as humidity and heat. Ridges run smoothly, at certain regions they form distinctive shapes characterized by ridge terminations and high curvature. Those regions are known as singularities or singular regions. Ridge physical characteristics exhibit minute patterns, with few examples as such: the ridge becomes discontinuous (ridge-ending), ridge splits into two (bi-furcation), and short ridge. These physical characteristics are common; however, they are in unique combinations for every fingerprint and are known as minutiae (see Fig. 1). The features can be classified between *i.e.,* high or low level (Tahmasebi & Kasaei, 2002; Jan et al., 2016), the former singular region and latter minutiae (Maio & Maltoni, 1997).

Ridges and valleys exhibit oriented sinusoidal-shaped patterns which is a fundamental property in the thorough analysis of fingerprint images. The local ridge orientation of each ridge pixel denoted by [*x*, *y*], is the angle ridge forms crossing through an arbitrarily small neighbourhood of non-overlapping blocks, centered at [*x*, *y*] with the horizontal axis. The dominant direction to which the ridges are oriented is taken as the orientation of the block. Another useful property is ridge frequency which is, at a point [*x*, *y*], the number of ridges per unit length along a non-overlapping block centered at [*x*, *y*] and orthogonal to orientation. In short, it is the distance between ridges at each point in a local non-overlapping window neighbourhood in fingerprint processing.

An individual can have one type of pattern or a mix of the patterns, however, the patterns are unique and they do not change over time. No two individuals have been found to have the same finger pattern (Treisman, 1985). Hence of individuality, the ease of studying, and its non-invasive and inexpensive methods of capturing, fingerprints are the go-to metric for biometric identification in the past century. Optical sensors and solid sensors capture fingerprint images, each with its own merits. In optical sensors, a finger is placed onto a surface and visible light is directed at it. The valleys reflect back the light while the ridges absorb the light, hence enabling one to differentiate between ridges, appearing dark, and valleys appearing bright. Solid state sensors, by applying pressure onto the sensor with a finger, measure and digitizes the physical shape. For a digital fingerprint image, the input is a matrix, containing values representing the pixel’s brightness intensity, and hence a representation of the original data captured by sensors. Additional image processing techniques are carried out to remove any sensor noise.

In fingerprint identification, minutiae from the fingerprint image are vital for the verification of an individual. The system identification performance is dependent on the accuracy and reliability of the features extracted. The fingerprint minutiae ridge pattern is matched with the corresponding minutiae *via* alignment and pairing for identification. Key features of a fingerprint from the fingerprint’s image may not be always easy to pick as identification depends on the image’s quality or a finger’s quality. In a good image ridges and valleys are patterned in a smooth manner, with the ease of distinguishing between the two. However, in a real scenario, image quality depends on the finger’s condition and the scanner’s ability to record a fingerprint. A finger’s skin affected by cuts bruises or if the skin’s surface is too wet/dry are a few factors that determine the quality of a fingerprint image. Natural abrasion of ridges *via* aging or manual labor results in difficulty in identifying ridge patterns. Apart from the physical condition of fingers, incorrect finger pressure technique and sensor noise are also factors a fingerprint image may be of poor quality. These issues may cause ridges to appear discontinuous making it look as if there are extra minutiae when there are not. Other issues include parallel ridges seeming as one, genuine minutiae features being corrupted or incorrect location recorded of minutiae *i.e.,* orientation and position.

### Related work

The goal of image enhancement algorithms or filters is to recover the recoverable regions, as well as clarify the ridge details, improve the contrast between ridges and valleys, mitigate/eliminate any unnecessary noise elements, isolating the area of interest in the greyscale images. It is crucial in all images involving feature detection, as indicated by the current work done in this area for easy identification of minutiae that are not properly visible.

**Figure 2:**
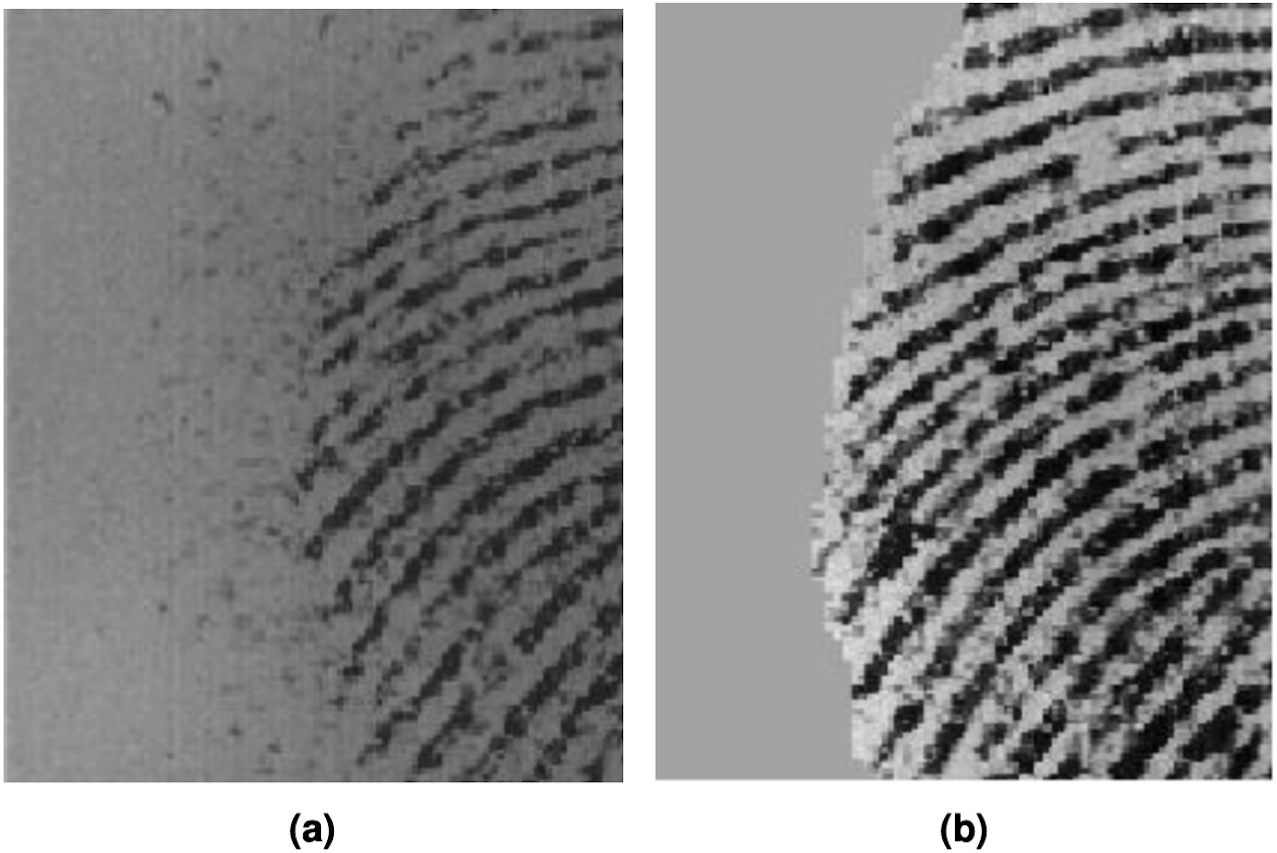
Fingerprint image (A) original (B) segmented.

Low-level systematic abstract operation on fingerprint images is image pre-processing. For a given pixel value of a fingerprint image, pre-processing transforms it to a newer value, while taking into account the nearby surroundings. They can be easily picked out and restored with respect to the neighboring pixels with pre-processing operations (Khalil et al., 2019). During fingerprint image analysis, a process called segmentation is carried out. Segmentation involves separating the fingerprint region from the image to ensure areas of the background do not interfere with the fingerprint’s features. It ensures no features are extracted from a noisy area within the background. The background of a fingerprint image is an isotropic pattern while the foreground consists of oriented stripes. Using a simple local intensity scheme, foreground and background can be distinguished from one another, provided the background is consistent and lighter than the foreground area. However, the introduction of noise from fingerprint scanners, dust specks, and grease require more robust techniques for segmentation. Foreground and background areas are distinguished from one another by using local histograms of ridge orientations, computing images into blocks, and estimating the orientation and histogram for each pixel (Mehtre et al., 1987; Chaudhary, Singh & Dimri, 2020). High peaks of a histogram indicate foreground area, while flat or near flat indicates isotropic region *i.e.,* the background region. On encountering a perfectly uniform area where local ridge orientation cannot be found, block variance can be used to differentiate the background/foreground of fingerprint images (Das, 2018). For each block, variance is calculated from greyscale values. Low variance values of blocks are denoted as the background of a fingerprint image while above a specified threshold is denoted as the foreground Fig. 2.

**Figure 3:**
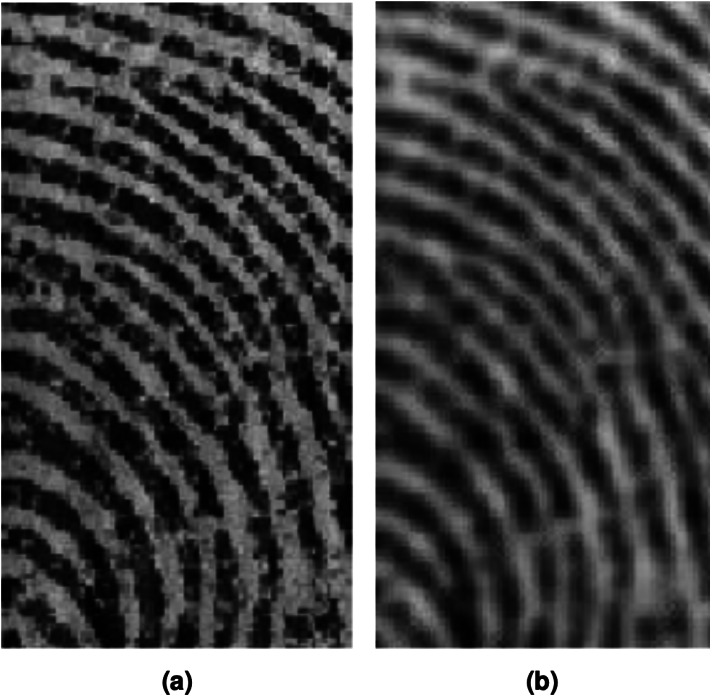
Fingerprint image (A) non-smoothed image (B) smoothed image.

Smoothing reduces noise and is a standard in pre-processing digital images. Many image processing methods do not perform well in a noisy environment (see Fig. 3), which is why smoothing is used as a preprocessing module in applications. The issue with traditional smoothing-based approaches to enhance fingerprint images and remove noise is the risk of smoothing relevant information within the image. The partial differential equation approach is among the preferred methods to achieve smoothing.

The fingerprint image is taken as an initial state of a parabolic diffusion process, wherein the diffusion is controlled by the derivative of the fingerprint image pixels *via* diffusion tensor, hence known as the coherence diffusion process which adaptively uses smoothing-based methods (Ali et al., 2012). It is based on the Perona & Malik (1990) model, in which the diffusion tensor enhances the coherent ridge structure of fingerprint images *via* a nonlinear diffusion process. The diffusion tensor controls the process of diffusion *i.e.,* smoothing at different directions (Liu et al., 2020). In other words, the algorithm treats each pixel according to the neighbouring pixels.

Gabor filters have the well-known property of being both frequency and orientation-selective with optimal combined resolution in different domains, thus achieving favorable and better performance (Xia, Lv & Sun, 2018). Gabor filters are defined by a sinusoidal plane wave, which itself is defined by its orientation and coordinates, incorporating a Gaussian envelope. Gabor filter’s parameters are tuned *i.e.,* the Gaussian envelope standard deviations, with respect to *x* and *y*. The higher the values more robust the filter at the cost of incurring spurious ridges, lower the values less likely to remove noise but not incur spurious ridges (Hajri et al., 2020). Despite the Gabor filter’s better performance, there are two limitations. The maximum bandwidth possible is approximately one octave, and the filter is not optimal in seeking widespread frequency information alongside maximum spatial localization. Also, it is to be noted that the signal orthogonal to the local region’s orientation cannot be always represented by a sinusoidal wave hence Gabor filter performance is impaired in bandwidth-oriented areas. An alternative solution is a log-Gabor filter, the filter is optimized as it is constructed with arbitrary bandwidth (Wang et al., 2008). This also reduces the over-representation of lower-ranged frequencies. An added advantage is the visual aspect of symmetric cell responses on the logarithmic frequency axis as a function of log-Gabor.

Log-Gabor has Gaussian functions, each viewed on the frequency and logarithmic frequency scales respectively. Given the singularity at the origin of the log function hence an analytic expression in the spatial image domain cannot be constructed, and due to their properties of frequency selection and orientation selection log-Gabor filter utilizes frequency representation. Log-Gabor filter in several applications has led to promising results *i.e.,* facial expression classification (Vatcharaphrueksadee et al., 2022), iris identification (Mukherjee et al., 2021; Jayavadivel & Prabaharan, 2021), finger knuckle enhancement (Benmalek et al., 2022). Log-Gabor filter is also used in textural feature detections for different applications such as cataract detection (Chande et al., 2022).

## Overview of Proposed Algorithm

**Figure 4:**
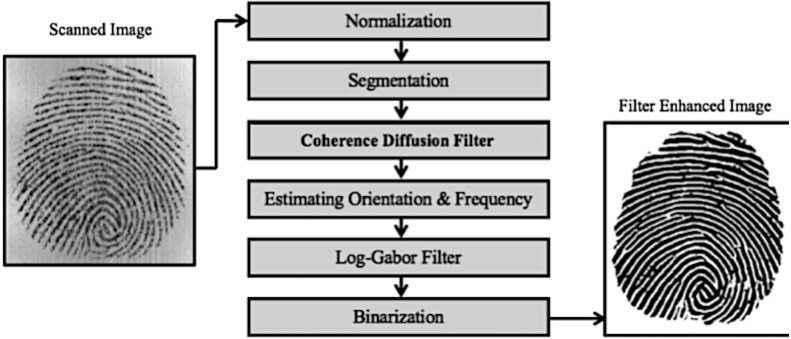
Proposed fingerprint enhancement filter.

This work uses a combination of two filters for fingerprint image enhancement. The initial step is normalization, which places the image greyscale intensity values in a pre-specified range of mean and variance. This allows actual intensity data values to be allotted more range of values, hence visually improving contrast and providing a wider range of intensity values to work with. An incorrect digital capture of a fingerprint image compromises the greyscale intensity values that are representing the fingerprint and the surrounding region. The surrounding region capture, *i.e.,* background, includes scanner noise and specks that effects minutiae detection algorithms hence the foreground region of interest is separated from the background *via* segmentation. To remove noise and specks within the foreground region of interest, traditional image processing uses a Gaussian low pass filter that softens edges as well as other surface irregularities during image capture at the cost of finer detail and features. Figure 4 represents the overall process.

In the proposed work, the image is passed through a coherence diffusion filter. The filter on detecting ridges controls the smoothing *via* the diffusion tensor, lesser smoothing process at edges, and more smoothing process at non-edges. Before the fingerprint image can be passed through log-Gabor filter enhancement, the filter parameters need to be tuned to the fingerprint image’s intrinsic ridge structure properties. Once the fingerprint’s ridge structural orientation and frequency that make up the ridge pattern is determined, then the fingerprint image is passed through the log-Gabor filter. Ridges can be enhanced by the log-Gabor filter instead of a Gabor filter due to its added advantage of increased bandwidth and non-overrepresentation of lower frequencies. This is useful in regions where ridges exhibit sharp curvature. Log-Gabor filter greyscale output is converted to black and white for better feature detection and visual contrast through a process of binarization *via* Otsu thresholding.

## Filter Analysis and Design

### Normalization

Taking the greylevel values of pixel (*i*, *j*) from a fingerprint image *f*(*i*, *j*), within *N* × *N* window, the following steps achieve the normalization:

1. Calculate the mean of the fingerprint image
  (1) \begin{eqnarray*}\text{mean}= \frac{1}{{N}^{2}} \sum _{i=0}^{N-1}\sum _{j=0}^{N-1}f(i,j)\end{eqnarray*}
2. Calculate variance by
  (2) \begin{eqnarray*}\text{Var}= \frac{1}{{N}^{2}} \sum _{i=0}^{N-1}\sum _{j=0}^{N-1}{ \left( f(i,j)-\text{mean}(I) \right) }^{2}\end{eqnarray*}
3. Fingerprint image pixel intensity values are normalized by the following process
  (3) \begin{eqnarray*}G(i,j)= \left\{ \begin{array}{@{}l@{}} \displaystyle {\text{mean}}_{0}+\sqrt{ \frac{{\text{var}}_{0}(I(i,j)-\text{mean})^{2}}{\text{var}} }; \text{if} f(i,j)\gt M \\ \displaystyle {\text{mean}}_{0}+\sqrt{ \frac{{\text{var}}_{0}(I(i,j)-\text{mean})^{2}}{\text{var}} }; \text{otherwise} \end{array} \right. \end{eqnarray*}

where mean_0_ and var_0_ are desired respective mean and variance values to which the newer normalized pixel intensity *i.e., G*(*i*, *j*) is equally spread around. This ensures better utilization of pixel intensity values with better contrast.

### Segmentation

The foreground is a pattern made up of black and white regions, representing ridges and valleys. Hence, the foreground region will show higher variance when compared to the background regions. Taking the greylevel intensity values from a fingerprint image *g*(*i*, *j*), defined by *w* × *w* window, at pixel (*i*, *j*), following steps achieves segmentation:

1. Divide image fingerprint image into blocks, of *w* × *w* pixels (*w* is taken as 16). Calculate the mean value of each block *via*
  (4) \begin{eqnarray*}\text{mean}(m,n)=\sum _{w}g(i,j)\end{eqnarray*}
  where *g*(*i*, *j*) denotes the pixel grey value and *m*, *n* represents the image’s respective block’s row and column.
2. Calculate each of the block’s variance values
  (5) \begin{eqnarray*}\text{var}(m,n)= \frac{\sum _{i=1}^{w}\sum _{j=1}^{w}[g(i,j)-\text{mean}]^{2}}{w\times w} \end{eqnarray*}
  where *m* = 1, 2, …, *M* and *n* = 1, 2, …, *N*.
3. Check if the variance is above a certain threshold *T* for each block
  (6) \begin{eqnarray*}\text{var}(m,n)= \left\{ \begin{array}{@{}l@{}} \displaystyle \text{var}(m,n);\text{var}(m,n)\gt T \\ \displaystyle 0 ;\text{otherwise} \end{array} \right. \end{eqnarray*}

This leaves behind the area of interest *i.e.,* the foreground.

### Coherence diffusion filter

A coherence diffusion filter is a nonlinear diffusion enhancement algorithm consisting of partial differential equations which retain fingerprint intensity image values based on the gradient of neighbouring image intensities. The filter smooths the images adaptively to its requirements.

(7) \begin{eqnarray*}{\delta }_{t}I=\text{div}(\text{D}.\nabla I)\end{eqnarray*}

and

(8) \begin{eqnarray*}\text{D}= \frac{1}{1+{ \left( \frac{{|}{|}\nabla {|}{|}}{k} \right) }^{2}} \end{eqnarray*}

where “div” is the divergence operator, “D” is diffusion tensor, “ ∇*I*” is image gradient and “ *k*” is the respective constant.

### Log-Gabor filter

The log-Gabor filter is two parts, the radial filter frequency response and angular frequency response as shown below

(9) \begin{eqnarray*}{\text{G}}_{r}(r)=\exp \nolimits \left( - \frac{[\log \nolimits ( \frac{r}{{f}_{\circ }} )]^{2}}{2.{\sigma }_{r}^{2}} \right) \end{eqnarray*}

(10) \begin{eqnarray*}{\text{G}}_{\theta }(\theta )=\exp \nolimits \left( - \frac{[\theta -{\theta }_{0}]^{2}}{2.{\sigma }_{\theta }^{2}} \right) \end{eqnarray*}

Multiplying them together provides us with the log-Gabor filter through which the fingerprint is passed.

(11) \begin{eqnarray*}\text{G}(r,\theta )={\text{G}}_{r}(r).{\text{G}}_{\theta }(\theta )\end{eqnarray*}

Polar coordinate (*r*, *θ*), the local center frequency *f*_0_, the local orientation angle *θ*_0_, the scale bandwidth *σ*_*r*_ and angular bandwidth *σ*_*θ*_ is used in the construction of the log-Gabor filter. These four parameters are used to control the filter’s response. The orientation angle and the frequency field are calculated by the steps mentioned below, both values are instantaneous in nature to the local region of a fingerprint image. The following steps calculate the orientation angle *θ*_0_ and center frequency *f*_0_ for the local region.

1. Divide image into equal blocks, each of size B × B. For a 500 dpi resolution of a fingerprint image, the average frequency of fingerprint ridges (distance between two ridges) is 8 pixels. Hence image blocks twice the frequency size (16 × 16) will contain relevant ridge structure (Stojanović et al., 2016). For block sizes lesser than *w*, the computation time increases while the clarity of features degrades when the block size is increased above *w*.
2. Calculate gradients *δ*_*x*_(*x*, *y*) and *δ*_*y*_(*x*, *y*) of each pixel within the block.
3. Calculate the orientation of each block at pixel (*i*, *j*)
  (12) \begin{eqnarray*}{\theta }_{0}= \frac{1}{2} {\tan \nolimits }^{-1} \left( \frac{\sum _{u=i- \frac{W}{2} }^{i+ \frac{W}{2} }\sum _{v=j- \frac{W}{2} }^{j+ \frac{W}{2} }2{\delta }_{x}(u,v){\delta }_{y}(u,v)}{\sum _{u=i- \frac{W}{2} }^{i+ \frac{W}{2} }\sum _{v=j- \frac{W}{2} }^{j+ \frac{W}{2} }{\delta }_{x}^{2}(u,v)-{\delta }_{y}^{2}(u,v)} \right) \end{eqnarray*}
4. The orientation is orthogonal in the frequency domain to the spatial domain, hence
  (13) \begin{eqnarray*}{\theta }_{0}={\theta }_{0}+ \frac{\pi }{2} \end{eqnarray*}
  to get the corresponding angle in the frequency domain.
5. Within each block, take pixel values that are orthogonal to the local orientation. From the resulting *B* × *B* matrix of pixel values, summing each column will result in a one-dimensional array *i.e.,* a one-dimensional *x*-signature wave with extrema representing ridge and valleys waveform (Orczyk & Wieclaw, 2011).

The extrema correspond to the pattern of the fingerprint image. The frequency *f*_0_ is calculated from the one-dimensional wave *via*

**Figure 5:**
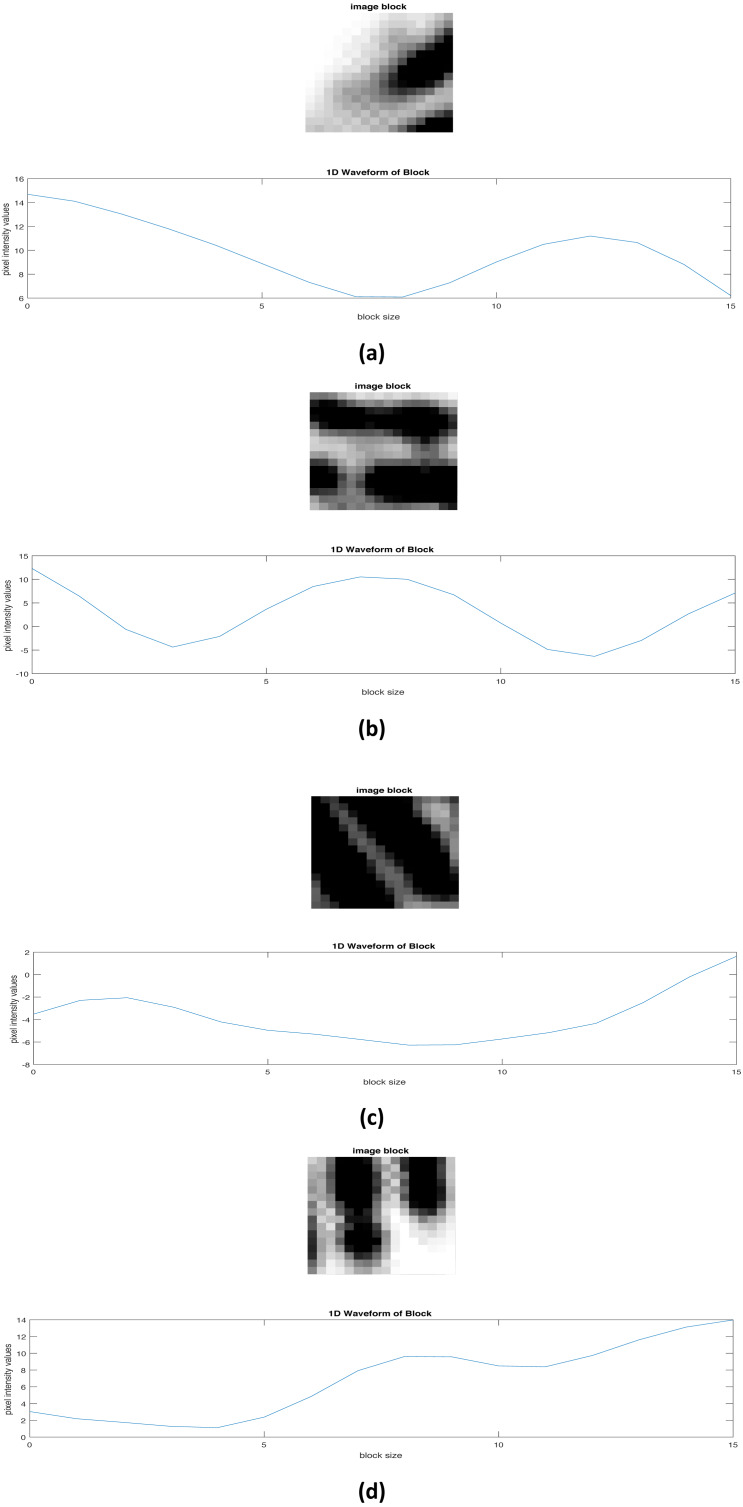
Ridge and valley 1D waveform in given blocks of a fingerprint image.

(14) \begin{eqnarray*}{f}_{0}= \frac{1}{T(i,j)} \end{eqnarray*}

where *T*(*i*, *j*) is the total number of pixels given between consecutive ridges *i.e.,* peaks (see Fig. 5). Hence *θ*_0_ and *f*_0_ corresponds to equations (9) and (10), respectively.

### Binarization

The resulting fingerprint image is composed of different greyscale pixel intensity values. For better contrast enabling more clarity of ridges and valleys binarization is recommended to improve contrast *via* Otsu thresholding. A local threshold *T* is calculated, and any pixel value of a fingerprint image above *T* is fixed to a value of one while below the threshold is set as zero. Finding the threshold *T* involves the minimization of intra-class variances

(15) \begin{eqnarray*}{\sigma }_{w}^{2}(t)={w}_{0}(t){\sigma }_{0}^{2}(t)+{w}_{1}(t){\sigma }_{1}^{2}(t)\end{eqnarray*}

where *w*_0_ and *w*_1_ are the probabilities of two classes, with threshold *t* separating them apart. Variances of the two classes are denoted by ${\sigma }_{0}^{2}$ and ${\sigma }_{1}^{2}$.

(16) \begin{eqnarray*}{w}_{0}(t)=\sum _{i=0}^{t-1}p(i){w}_{1}(t)=\sum _{i=t}^{L-1}p(i)\end{eqnarray*}

Between intra-class variance and inter-class variance, reducing the former increases the latter and is denoted *via* the class’s probabilities *w* and mean µ.

(17) \begin{eqnarray*}{\sigma }_{b}^{2}(t)={w}_{0}(t){w}_{1}(t)[{\mu }_{0}(t)-{\mu }_{1}(t)]^{2}.\end{eqnarray*}

A greyscale image has 256 depth of pixel intensities, the threshold *T* value is selected that corresponds to the lowest class variance. Hence for a fingerprint image *f*(*i*, *j*)

(18) \begin{eqnarray*}f(i,j)= \left\{ \begin{array}{@{}l@{}} \displaystyle 1; f(i,j)\gt T \\ \displaystyle 0; \text{otherwise}. \end{array} \right. \end{eqnarray*}

## Experimental Results

**Figure 6:**
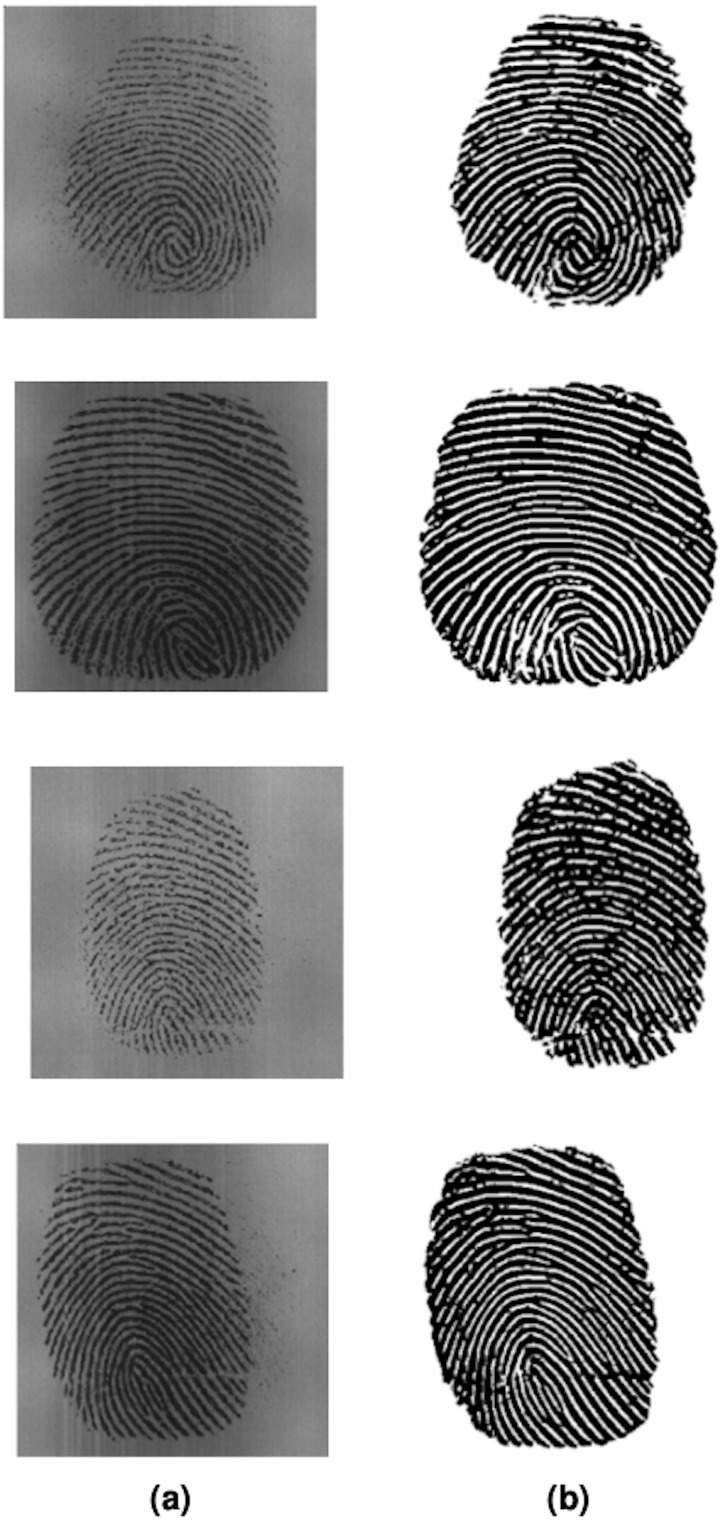
(A) FVC database original images. (B) Image enhanced *via* proposed method.

**Figure 7:**
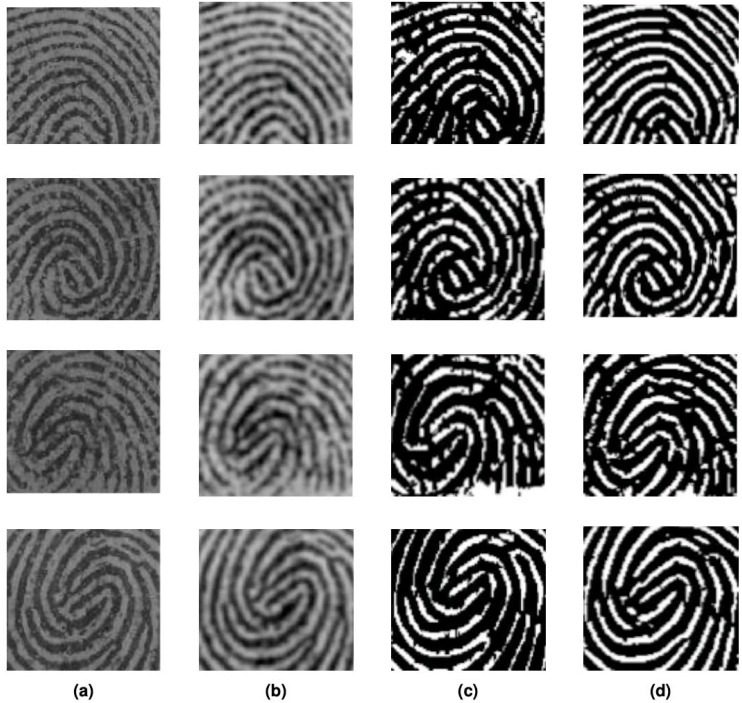
(A) FVC database original image, (B) coherence diffusion, (C) log-Gabor filter, (D) proposed method.

The proposed study was implemented in MATLAB on Fingerprint Verification Competition (FVC) image database. The database is established by the Biometric System Laboratory (University of Bologna), the U.S. National Biometric Test Center (San Jose State University), and the Pattern Recognition and Image-Processing Laboratory (Michigan State University). There are four released editions of the fingerprint images FVC database in the years 2000, 2002, 2004, and 2006 as benchmarks for testing and comparison of fingerprint images. Amongst the four databases, the proposed algorithm is implemented on the Fingerprint Verification Competition (2002) (FVC) fingerprint image database (Maltoni et al. (2002)). The database consists of fingerprint images from 10 volunteers and is divided into four subcategories DB1, DB2, DB3, and DB4. Each subcategory contains 80 fingerprint images, *i.e.,* each participant provides eight images per finger. DB1 consists of good-quality fingerprint images, while DB2 and DB4 are of adequate quality, with DB3 consists of images that are low quality. DB2 contains images of dimensions 388 × 374 while DB3 contains images of dimensions 300 × 300. To successfully extract minutiae from a fingerprint picture, an enhancement technique is necessary to increase the clarity of the original image’s ridges and valleys. The proposed filter was implemented on both DB2 and DB3 databases with promising results as shown in Fig. 6. Column (a) are the original FVC2002 DB2 and DB3 images while (b) shows the workings of the proposed algorithm on FVC2002 DB2 and DB3 images. Figure 7 shows Coherence-Diffusion & log-Gabor filter outputs side by side next to the proposed algorithm, and on the visual comparison, the proposed method shows higher clarity of ridges and valleys in comparison with stand-alone filters. Image enhancement on fingerprint images improves clarity between ridges and valleys and can aid in further processing such as minutiae detection. The ridges are not clear in either of the stand-alone filters, each showing noise and specks.

 The performance of the proposed filter was measured against the coherence diffusion filter and log-Gabor filter. The algorithms were tested on FVC2002 DB3′s very low-quality fingerprint image database. In order for two fingerprints to be considered from the same individual, a fixed number of minutiae are to match between the fingerprint images. The matching reliability and robustness are dependent on minutiae extraction accuracy, hence the importance of fingerprint image enhancement. The three minutiae errors that can affect matching are defined as such:

• Missing: genuine minutiae is not detected by the minutiae detection algorithm.
• Spurious: minutiae that are non-existent are detected by the minutiae detection algorithm.
• Exchanged: bifurcation is mistaken as ridge ending and vice versa by the minutiae detection algorithm.

The average error percentages of the minutiae extracted from the DB3 database, by each of the three filters, are shown in Table 1. Average error percentages of ridge bifurcation minutiae and ridge termination minutiae are noted for fingerprint images that have undergone enhancement, thus providing the total error percentage for each of the enhancement algorithms. There is a marked improvement in the performance of the proposed fingerprint image enhancement over the coherence diffusion filter and log-Gabor filter. It can be seen that the stand-alone coherence diffusion error rate is more than two times the error rate of the proposed solution. The stand-alone log-Gabor filter performance does not fare any better, as its error rate exceeds the proposed solution error rate.

**Table 1:** Average error percentages of the minutiae extracted from FVC 2002 database.

**Enhancement algorithm**	**Missing (%)**		**Spurious (%)**		**Exchanged (%)**		**Total error (%)**
	Bifurcation	Termination	Bifurcation	Termination	Bifurcation	Termination	
Coherence diffusion filter	9	35	16	6	2	5	73
Log-gabor filter	6	31	7	13	2	5	64
Proposed filter	2	19	5	2	0	7	35

## Discussion

There is improvement in clarity between ridges valleys, better minutiae visibility, and fewer noise specks after the application of the proposed algorithm. On visual comparison there is a marked improvement, the ridges and valleys are uniform, and the enhanced algorithm has led to better-quality of fingerprint images that can be used for identification purposes. In regions of high curvature in fingerprint images, there is higher clarity due to the coherence diffusion filter as compared to other traditional filters, while the whole structure is covered due to wider coverage of spectral information *via* the log-Gabor filter. The suggested enhancement filter provides better performance. The visual result shows the outputs of each method *i.e.,* the coherence diffusion filter, the log-Gabor filter, and our proposed method. There is higher clarity in the proposed filter output as compared to the stand-alone coherence diffusion filter and log-Gabor filter.

This is observed by the surrounding structure, there is less noise speck, and a clear distinction between ridges and valleys. Coherence-Diffusion & log-Gabor filter help reduce processing imperfections caused by ridge cracks, and false ridges, and mitigate noise and uneven lighting in the fingerprint. The stand-alone coherence diffusion filter provides enhancement at uniform ridge-valley areas while at non-uniform regions there is less enhancement. While the stand-alone log-Gabor provides an enhancement for the whole structure, at higher curvature, there is less enhancement. A combination of both filters covers enhancement for the whole fingerprint image structure. The quantitative assessment shows proposed method helps in the clear detection of minutiae as compared to stand-alone filters.

## Conclusion

The goal of the study is to highlight the importance of fingerprints in biometric identification, the issues prevailing on its usage, and an improved solution as well as implementation. Implementation of the proposed method on fingerprint images has led to promising results. The coherence Diffusion filter maintained the structure while smoothing which otherwise is lost in the application of traditional smoothing schemes. A traditional Gabor filter has bandwidth limitations and seeking a larger scale of spectral components is not possible. The workaround is an improvement on an existing solution by using Log-Gabor Filter. enabling a broad range of spectral information with limited DC Component interference. The result is higher clarity between ridges and valleys of fingerprint images easier to identify minutiae. The log-Gabor filter has increased bandwidth and is tuned to the smallest spatial extent possible. This unique feature significantly improves image quality when used for fingerprint enhancement. In practice, the Log-Gabor filter may effectively increase the contrast between ridges and valleys in fingerprint pictures while retaining the ridge structure. The 2D spatial domain log-Gabor filter is used to improve regions of low curvature ridge-valley pattern, with uniform orientation and spacing. The output images of each filter are combined to produce the final enhanced image. Experimental visual and quantitative analysis results indicate that the suggested enhancement technique effectively improves the quality of fingerprint images and increases the reliability of automatic fingerprint identification systems.

## Supplemental Information

10.7717/peerj-cs.1183/supp-1 Supplemental Information 1 Matlab Code

## Additional Information and Declarations

### Data Availability

The following information was supplied regarding data availability: The raw data and code is available in the Supplemental Files. Peter Kovesi provided guidance for Matlab Code which is available at: https://www.peterkovesi.com/matlabfns/#phasecong (Kovesi, 2000). The dataset is available at FVC2002: http://bias.csr.unibo.it/fvc2002/download.asp

## References

[ref-1] Ali A, Jing X, Jie Z, Saleem N (2012). Fingerprint image enhancement using coherence diffusion filter and gabor filter. Journal of Informational & Computational Science.

[ref-2] Benmalek M, Attia A, Bouziane A, Hassaballah M (2022). A semi-supervised deep rule-based classifier for robust finger knuckle-print verification. Evolving Systems.

[ref-3] Bhatti DMS, Khalil RA, Saeed N, Nam H (2022). Detection and spatial correlation analysis of infectious diseases using wireless body area network under imperfect wireless channel. Big Data.

[ref-4] Chande K, Jha P, Aulakh KK, Shinde S (2022). Cataract detection using textural features and machine learning algorithms.

[ref-5] Chaudhary N, Singh HP, Dimri P, Hu YC, Tiwari S, Trivedi M, Mishra K (2020). Comparative study of latent fingerprint image segmentation techniques based on literature review. Ambient Communications and Computer Systems. Advances in Intelligent Systems and Computing.

[ref-6] Das D (2018). A fingerprint segmentation scheme based on adaptive threshold estimation.

[ref-7] Datta AK, Lee HC, Ramotowski R, Gaensslen R (2001). Advances in fingerprint technology.

[ref-8] Hajri S, Kallel F, Hamida AB, Nait-Ali A (2020). A comparative study of fingerprint enhancement algorithms.

[ref-9] Jain A, Pankanti S (2001). Automated fingerprint identification and.

[ref-10] Jain AK, Ross A, Prabhakar S (2004). An introduction to biometric recognition. IEEE Transactions on Circuits and Systems for Video Technology.

[ref-11] Jan T, Zafar H, Khalil R, Ashraf M (2016). A blind source separation approach based on IVA for convolutive speech mixtures.

[ref-12] Jayavadivel R, Prabaharan P (2021). Investigation on automated surveillance monitoring for human identification and recognition using face and iris biometric. Journal of Ambient Intelligence and Humanized Computing.

[ref-13] Khalil R, Ashraf S, Jan T, Jehangir A, Khan J (2015). Enhancement of speech signals using multiple statistical models. Sindh University Research Journal-SURJ (Science Series).

[ref-14] Khalil RA, Jones E, Babar MI, Jan T, Zafar MH, Alhussain T (2019). Speech emotion recognition using deep learning techniques: a review. IEEE Access.

[ref-15] Kovesi PD (2000). MATLAB and octave functions for computer vision and image processing. https://www.peterkovesi.com/matlabfns.

[ref-16] Liu Z, Cao H, Zhang H, Lai J (2020). A fingerprint image enhancement method based on anisotropic diffusion and shock filtering.

[ref-17] Maio D, Maltoni D (1997). Direct gray-scale minutiae detection in fingerprints. IEEE Transactions on Pattern Analysis and Machine Intelligence.

[ref-18] Maltoni D, Maio D, Jain AK, Feng J (2002). FVC2002 fingerprint verification competition. http://bias.csr.unibo.it/fvc2002/download.asp.

[ref-19] Mehtre BM, Murthy NN, Kapoor S, Chatterjee B (1987). Segmentation of fingerprint images using the directional image. Pattern Recognition.

[ref-20] Merriam-Webster (2022). Merriam-Webster definition of biometrics. https://www.merriam-webster.com/dictionary/biometrics.

[ref-21] Mukherjee A, Islam MZ, Mamun-Al-Imran G, Ali LE (2021). Iris recognition using wavelet features and various distance based classification.

[ref-22] Noor K, Jan T, Basheri M, Ali A, Khalil RA, Zafar MH, Ashraf M, Babar MI, Shah SW (2018). Performances enhancement of fingerprint recognition system using classifiers. IEEE Access.

[ref-23] Orczyk T, Wieclaw L (2011). Fingerprint ridges frequency.

[ref-24] Perona P, Malik J (1990). Scale-space and edge detection using anisotropic diffusion. IEEE Transactions on Pattern Analysis and Machine Intelligence.

[ref-25] Stojanović B, Marques O, Nešković A, Puzović S (2016). Fingerprint ROI segmentation based on deep learning.

[ref-26] Tahmasebi AM, Kasaei S (2002). A novel adaptive approach to fingerprint enhancement filter design. Signal Processing: Image Communication.

[ref-27] Treisman A (1985). Preattentive processing in vision. Computer Vision, Graphics, and Image Processing.

[ref-28] Vatcharaphrueksadee A, Maliyaem M, Viboonpanich R, Phuangkamnerd S (2022). Facial emotion classification of multi-type datasets based on SVM classifier.

[ref-29] Wang W, Li J, Huang F, Feng H (2008). Design and implementation of Log-Gabor filter in fingerprint image enhancement. Pattern Recognition Letters.

[ref-30] Xia Z, Lv R, Sun X (2018). Rotation-invariant Weber pattern and Gabor feature for fingerprint liveness detection. Multimedia Tools and Applications.

