# Supplementary figures and images for: Fingerprint image enhancement using multiple filters (part 1 of 2)
Source: PeerJ Comput Sci. 2023 Jan 3;9:e1183. doi: 10.7717/peerj-cs.1183 (PMC10280261; doi:10.7717/peerj-cs.1183)

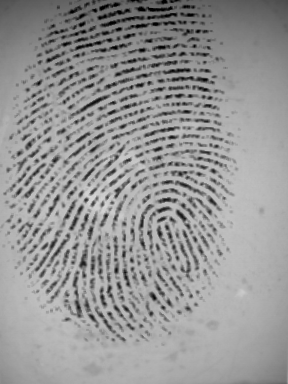

Supplement: Supplemental Information 1 [file peerj-cs-09-1183-s001.zip › FinalCode/Dataset/DB4_B/102_6.tif]

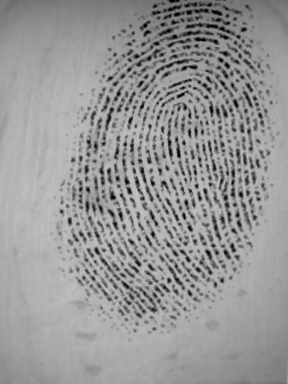

Supplement: Supplemental Information 1 [file peerj-cs-09-1183-s001.zip › FinalCode/Dataset/DB4_B/106_2.tif]

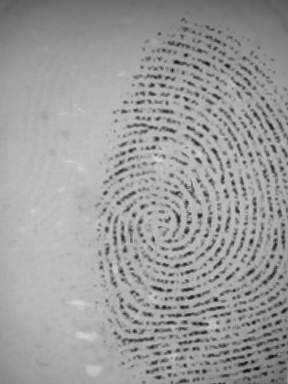

Supplement: Supplemental Information 1 [file peerj-cs-09-1183-s001.zip › FinalCode/Dataset/DB4_B/104_1.tif]

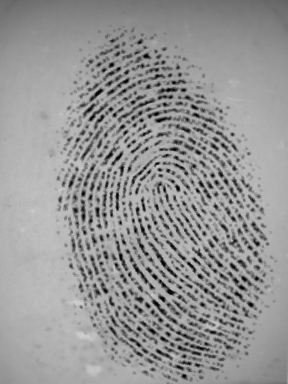

Supplement: Supplemental Information 1 [file peerj-cs-09-1183-s001.zip › FinalCode/Dataset/DB4_B/106_3.tif]

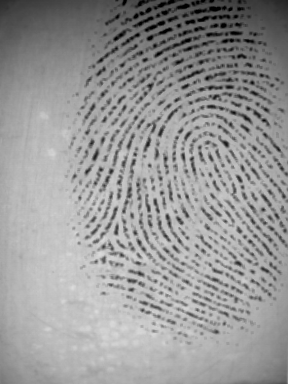

Supplement: Supplemental Information 1 [file peerj-cs-09-1183-s001.zip › FinalCode/Dataset/DB4_B/102_7.tif]

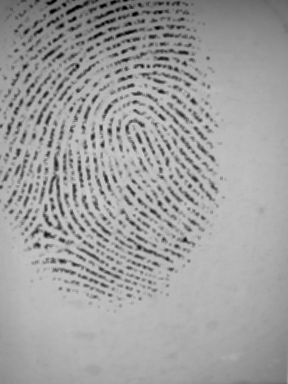

Supplement: Supplemental Information 1 [file peerj-cs-09-1183-s001.zip › FinalCode/Dataset/DB4_B/102_5.tif]

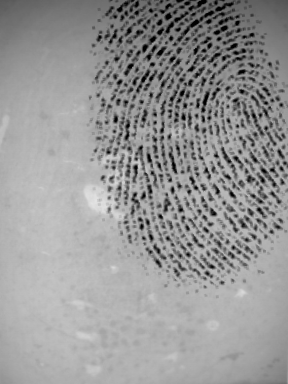

Supplement: Supplemental Information 1 [file peerj-cs-09-1183-s001.zip › FinalCode/Dataset/DB4_B/106_1.tif]

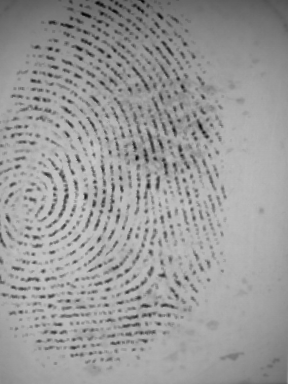

Supplement: Supplemental Information 1 [file peerj-cs-09-1183-s001.zip › FinalCode/Dataset/DB4_B/104_3.tif]

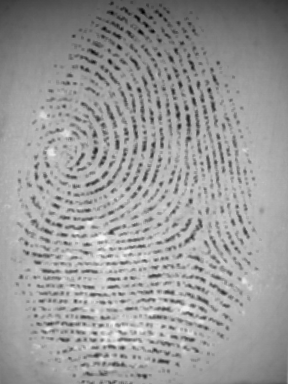

Supplement: Supplemental Information 1 [file peerj-cs-09-1183-s001.zip › FinalCode/Dataset/DB4_B/104_2.tif]

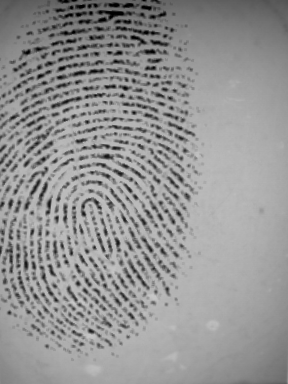

Supplement: Supplemental Information 1 [file peerj-cs-09-1183-s001.zip › FinalCode/Dataset/DB4_B/102_4.tif]

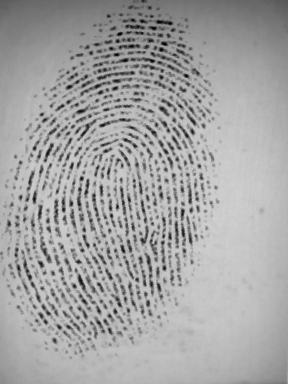

Supplement: Supplemental Information 1 [file peerj-cs-09-1183-s001.zip › FinalCode/Dataset/DB4_B/106_4.tif]

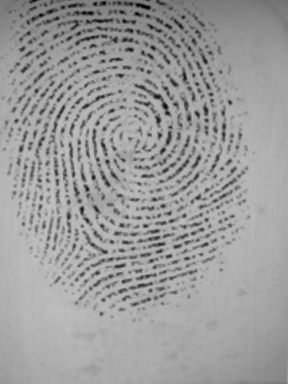

Supplement: Supplemental Information 1 [file peerj-cs-09-1183-s001.zip › FinalCode/Dataset/DB4_B/104_6.tif]

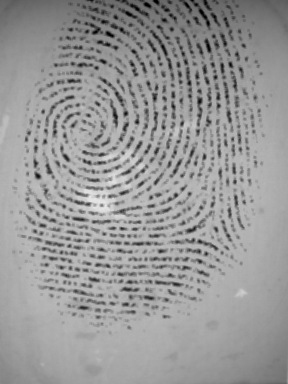

Supplement: Supplemental Information 1 [file peerj-cs-09-1183-s001.zip › FinalCode/Dataset/DB4_B/104_7.tif]

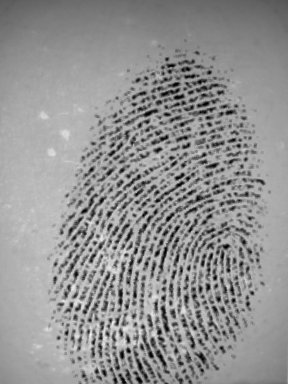

Supplement: Supplemental Information 1 [file peerj-cs-09-1183-s001.zip › FinalCode/Dataset/DB4_B/106_5.tif]

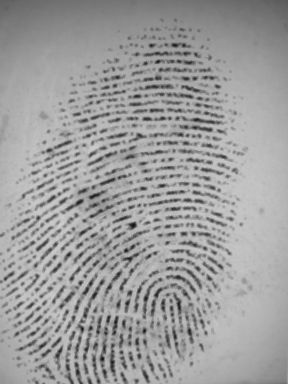

Supplement: Supplemental Information 1 [file peerj-cs-09-1183-s001.zip › FinalCode/Dataset/DB4_B/102_1.tif]

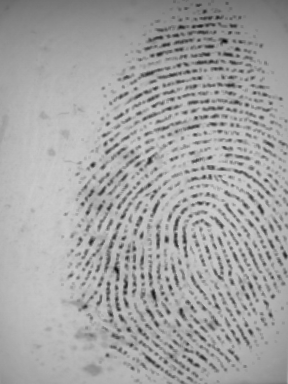

Supplement: Supplemental Information 1 [file peerj-cs-09-1183-s001.zip › FinalCode/Dataset/DB4_B/102_3.tif]

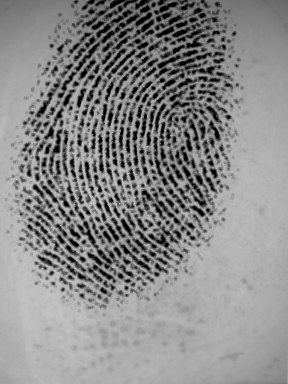

Supplement: Supplemental Information 1 [file peerj-cs-09-1183-s001.zip › FinalCode/Dataset/DB4_B/108_8.tif]

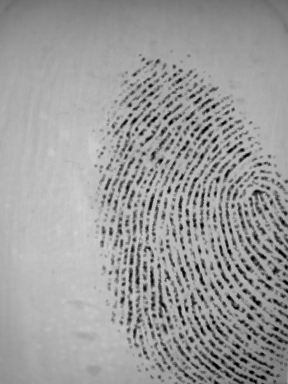

Supplement: Supplemental Information 1 [file peerj-cs-09-1183-s001.zip › FinalCode/Dataset/DB4_B/106_7.tif]

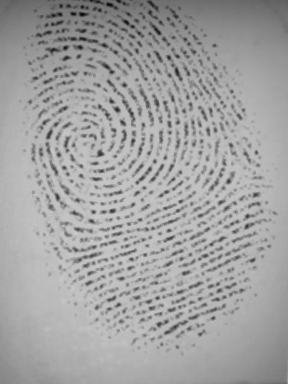

Supplement: Supplemental Information 1 [file peerj-cs-09-1183-s001.zip › FinalCode/Dataset/DB4_B/104_5.tif]

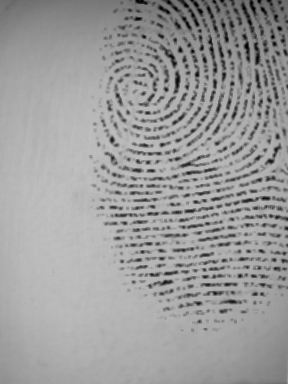

Supplement: Supplemental Information 1 [file peerj-cs-09-1183-s001.zip › FinalCode/Dataset/DB4_B/104_4.tif]

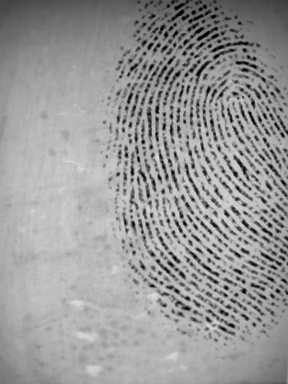

Supplement: Supplemental Information 1 [file peerj-cs-09-1183-s001.zip › FinalCode/Dataset/DB4_B/106_6.tif]

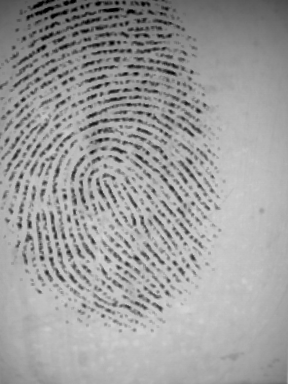

Supplement: Supplemental Information 1 [file peerj-cs-09-1183-s001.zip › FinalCode/Dataset/DB4_B/102_2.tif]

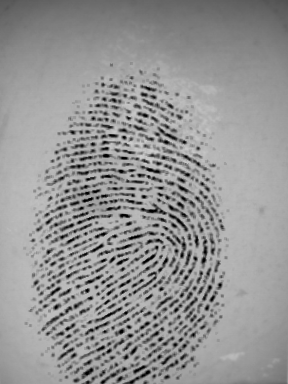

Supplement: Supplemental Information 1 [file peerj-cs-09-1183-s001.zip › FinalCode/Dataset/DB4_B/105_3.tif]

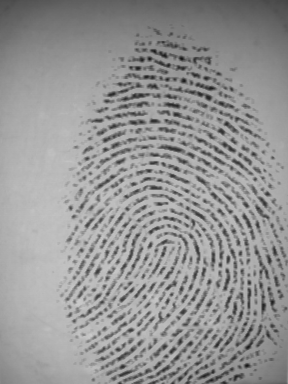

Supplement: Supplemental Information 1 [file peerj-cs-09-1183-s001.zip › FinalCode/Dataset/DB4_B/107_1.tif]

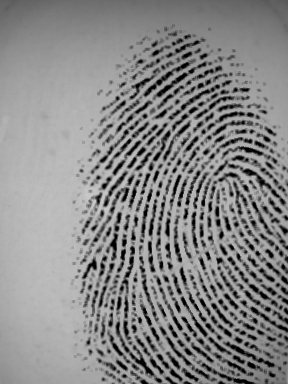

Supplement: Supplemental Information 1 [file peerj-cs-09-1183-s001.zip › FinalCode/Dataset/DB4_B/101_7.tif]

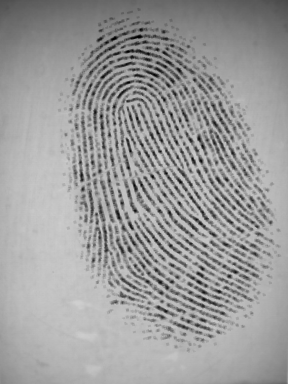

Supplement: Supplemental Information 1 [file peerj-cs-09-1183-s001.zip › FinalCode/Dataset/DB4_B/103_5.tif]

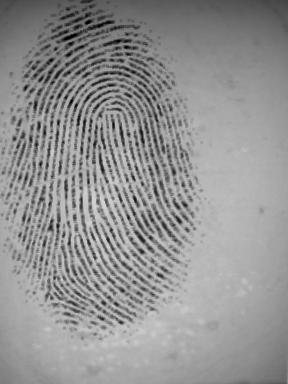

Supplement: Supplemental Information 1 [file peerj-cs-09-1183-s001.zip › FinalCode/Dataset/DB4_B/103_4.tif]

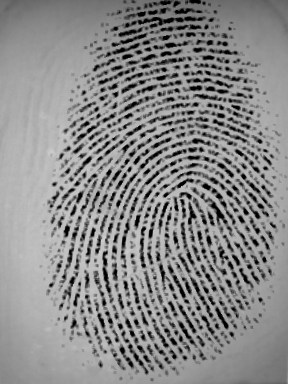

Supplement: Supplemental Information 1 [file peerj-cs-09-1183-s001.zip › FinalCode/Dataset/DB4_B/101_6.tif]

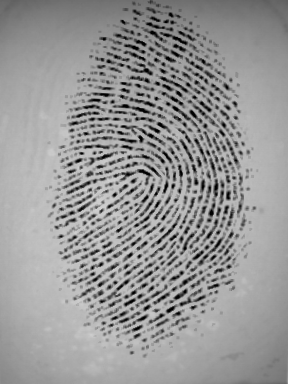

Supplement: Supplemental Information 1 [file peerj-cs-09-1183-s001.zip › FinalCode/Dataset/DB4_B/105_2.tif]

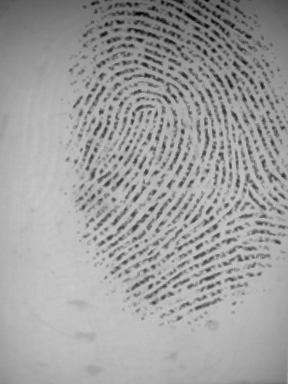

Supplement: Supplemental Information 1 [file peerj-cs-09-1183-s001.zip › FinalCode/Dataset/DB4_B/107_2.tif]

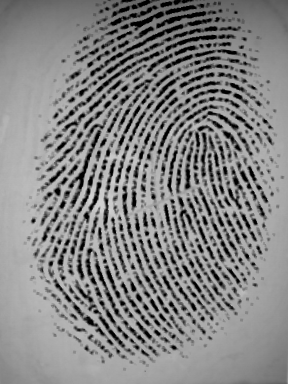

Supplement: Supplemental Information 1 [file peerj-cs-09-1183-s001.zip › FinalCode/Dataset/DB4_B/101_4.tif]

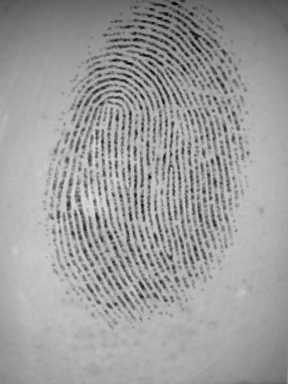

Supplement: Supplemental Information 1 [file peerj-cs-09-1183-s001.zip › FinalCode/Dataset/DB4_B/103_6.tif]

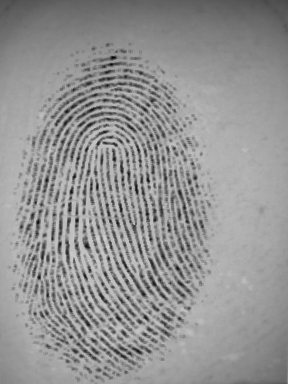

Supplement: Supplemental Information 1 [file peerj-cs-09-1183-s001.zip › FinalCode/Dataset/DB4_B/103_7.tif]

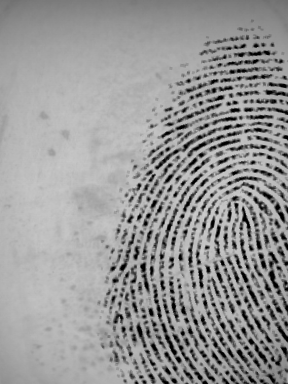

Supplement: Supplemental Information 1 [file peerj-cs-09-1183-s001.zip › FinalCode/Dataset/DB4_B/101_5.tif]

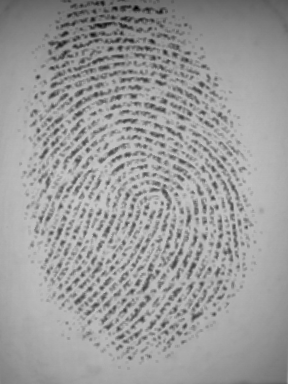

Supplement: Supplemental Information 1 [file peerj-cs-09-1183-s001.zip › FinalCode/Dataset/DB4_B/107_3.tif]

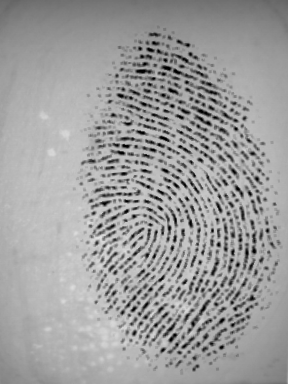

Supplement: Supplemental Information 1 [file peerj-cs-09-1183-s001.zip › FinalCode/Dataset/DB4_B/105_1.tif]

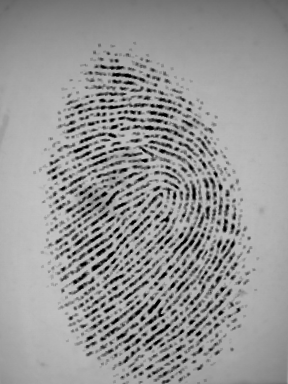

Supplement: Supplemental Information 1 [file peerj-cs-09-1183-s001.zip › FinalCode/Dataset/DB4_B/105_5.tif]

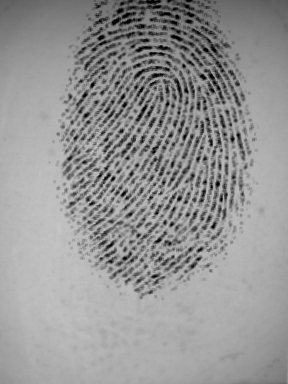

Supplement: Supplemental Information 1 [file peerj-cs-09-1183-s001.zip › FinalCode/Dataset/DB4_B/109_8.tif]

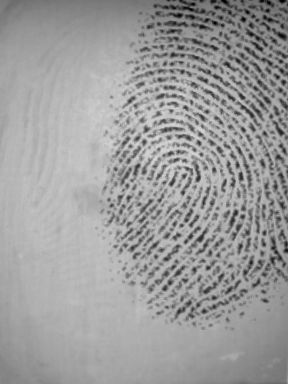

Supplement: Supplemental Information 1 [file peerj-cs-09-1183-s001.zip › FinalCode/Dataset/DB4_B/107_7.tif]

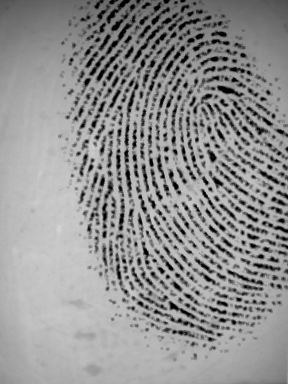

Supplement: Supplemental Information 1 [file peerj-cs-09-1183-s001.zip › FinalCode/Dataset/DB4_B/101_1.tif]

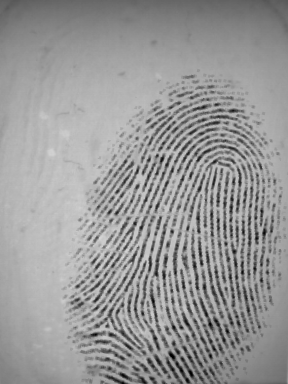

Supplement: Supplemental Information 1 [file peerj-cs-09-1183-s001.zip › FinalCode/Dataset/DB4_B/103_3.tif]

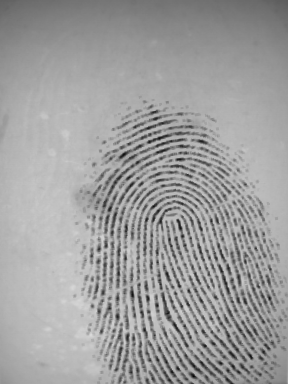

Supplement: Supplemental Information 1 [file peerj-cs-09-1183-s001.zip › FinalCode/Dataset/DB4_B/103_2.tif]

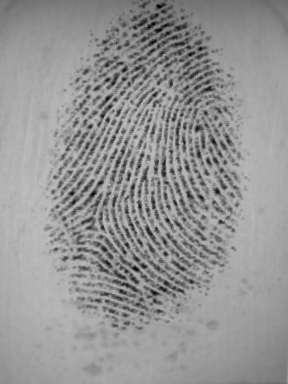

Supplement: Supplemental Information 1 [file peerj-cs-09-1183-s001.zip › FinalCode/Dataset/DB4_B/110_8.tif]

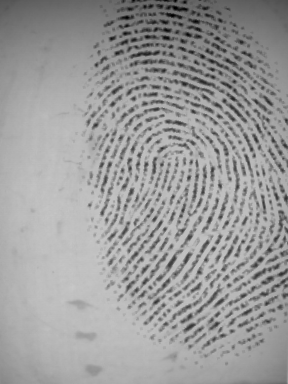

Supplement: Supplemental Information 1 [file peerj-cs-09-1183-s001.zip › FinalCode/Dataset/DB4_B/107_6.tif]

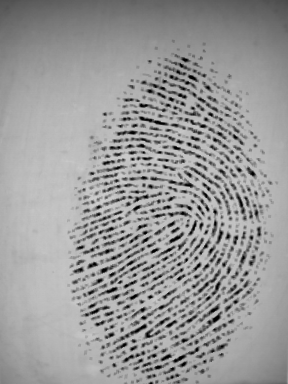

Supplement: Supplemental Information 1 [file peerj-cs-09-1183-s001.zip › FinalCode/Dataset/DB4_B/105_4.tif]

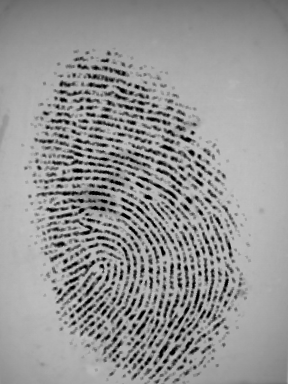

Supplement: Supplemental Information 1 [file peerj-cs-09-1183-s001.zip › FinalCode/Dataset/DB4_B/105_6.tif]

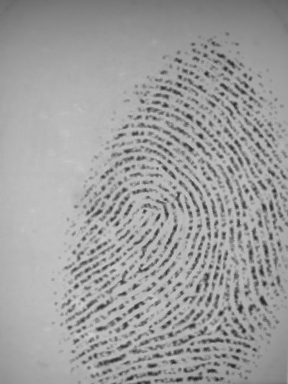

Supplement: Supplemental Information 1 [file peerj-cs-09-1183-s001.zip › FinalCode/Dataset/DB4_B/107_4.tif]

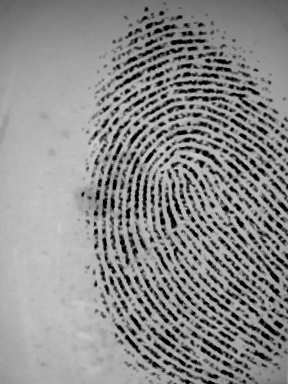

Supplement: Supplemental Information 1 [file peerj-cs-09-1183-s001.zip › FinalCode/Dataset/DB4_B/101_2.tif]

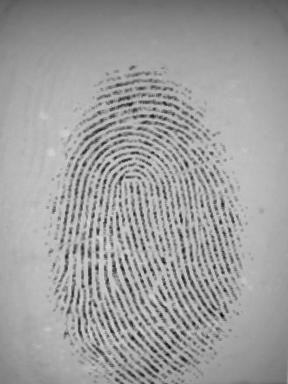

Supplement: Supplemental Information 1 [file peerj-cs-09-1183-s001.zip › FinalCode/Dataset/DB4_B/103_1.tif]

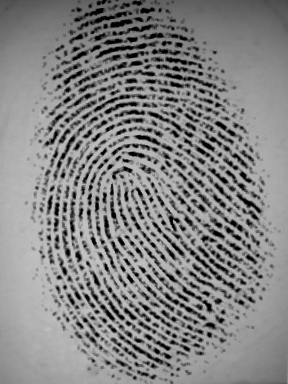

Supplement: Supplemental Information 1 [file peerj-cs-09-1183-s001.zip › FinalCode/Dataset/DB4_B/101_3.tif]

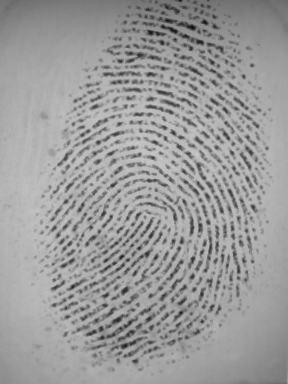

Supplement: Supplemental Information 1 [file peerj-cs-09-1183-s001.zip › FinalCode/Dataset/DB4_B/107_5.tif]

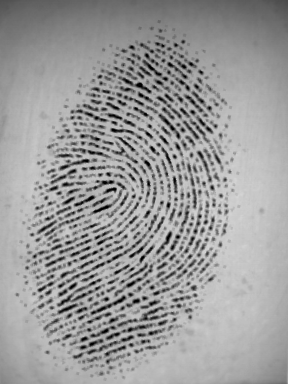

Supplement: Supplemental Information 1 [file peerj-cs-09-1183-s001.zip › FinalCode/Dataset/DB4_B/105_7.tif]

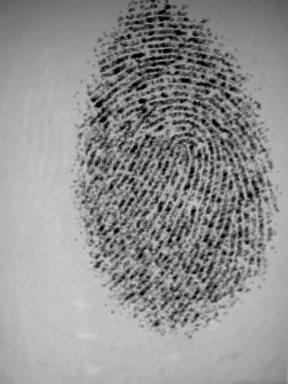

Supplement: Supplemental Information 1 [file peerj-cs-09-1183-s001.zip › FinalCode/Dataset/DB4_B/109_7.tif]

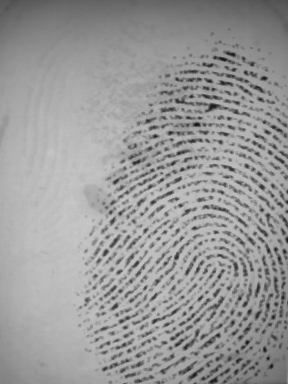

Supplement: Supplemental Information 1 [file peerj-cs-09-1183-s001.zip › FinalCode/Dataset/DB4_B/107_8.tif]

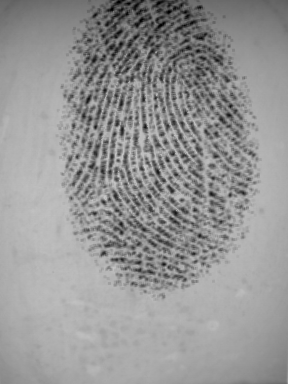

Supplement: Supplemental Information 1 [file peerj-cs-09-1183-s001.zip › FinalCode/Dataset/DB4_B/110_6.tif]

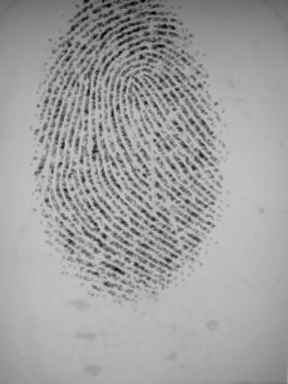

Supplement: Supplemental Information 1 [file peerj-cs-09-1183-s001.zip › FinalCode/Dataset/DB4_B/110_7.tif]

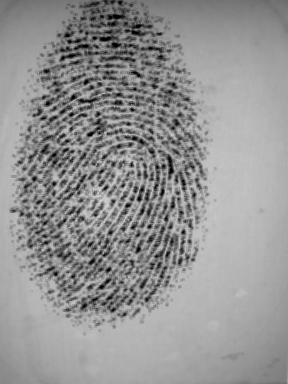

Supplement: Supplemental Information 1 [file peerj-cs-09-1183-s001.zip › FinalCode/Dataset/DB4_B/109_6.tif]

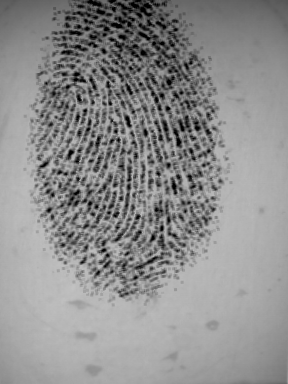

Supplement: Supplemental Information 1 [file peerj-cs-09-1183-s001.zip › FinalCode/Dataset/DB4_B/109_4.tif]

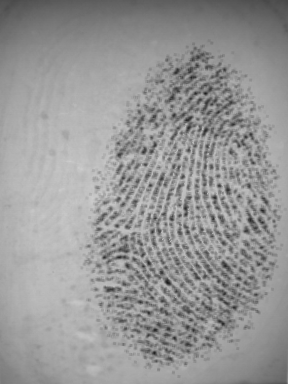

Supplement: Supplemental Information 1 [file peerj-cs-09-1183-s001.zip › FinalCode/Dataset/DB4_B/110_5.tif]

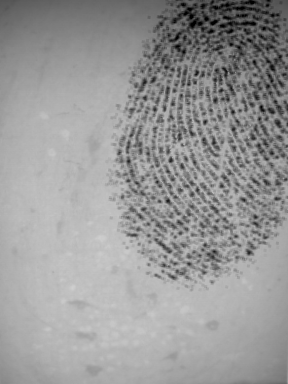

Supplement: Supplemental Information 1 [file peerj-cs-09-1183-s001.zip › FinalCode/Dataset/DB4_B/110_4.tif]

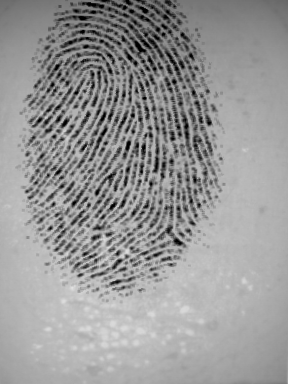

Supplement: Supplemental Information 1 [file peerj-cs-09-1183-s001.zip › FinalCode/Dataset/DB4_B/109_5.tif]

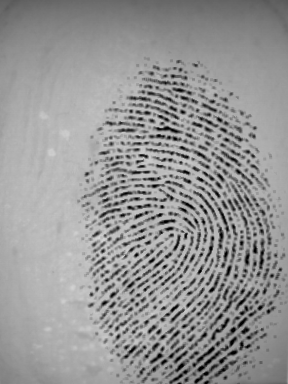

Supplement: Supplemental Information 1 [file peerj-cs-09-1183-s001.zip › FinalCode/Dataset/DB4_B/105_8.tif]

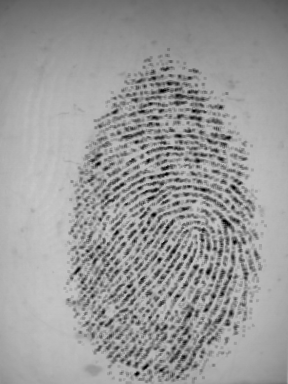

Supplement: Supplemental Information 1 [file peerj-cs-09-1183-s001.zip › FinalCode/Dataset/DB4_B/109_1.tif]

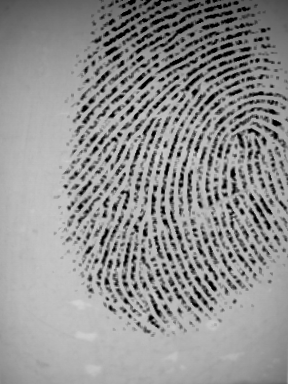

Supplement: Supplemental Information 1 [file peerj-cs-09-1183-s001.zip › FinalCode/Dataset/DB4_B/101_8.tif]

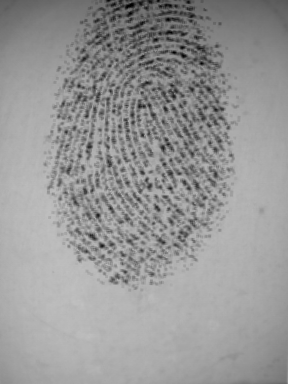

Supplement: Supplemental Information 1 [file peerj-cs-09-1183-s001.zip › FinalCode/Dataset/DB4_B/110_1.tif]

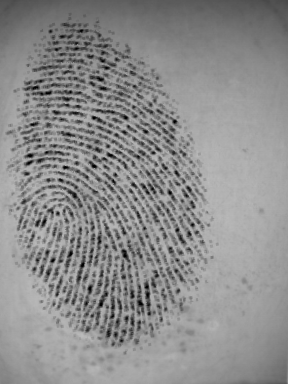

Supplement: Supplemental Information 1 [file peerj-cs-09-1183-s001.zip › FinalCode/Dataset/DB4_B/109_2.tif]

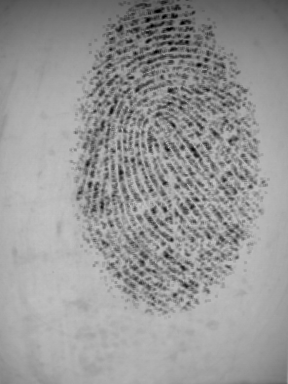

Supplement: Supplemental Information 1 [file peerj-cs-09-1183-s001.zip › FinalCode/Dataset/DB4_B/110_3.tif]

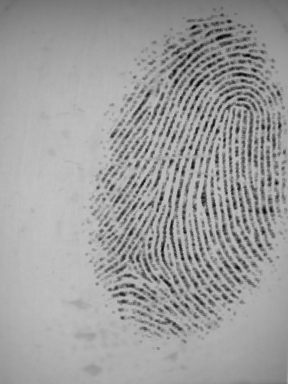

Supplement: Supplemental Information 1 [file peerj-cs-09-1183-s001.zip › FinalCode/Dataset/DB4_B/103_8.tif]

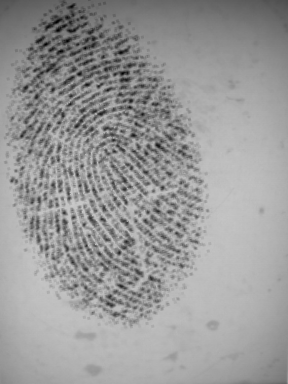

Supplement: Supplemental Information 1 [file peerj-cs-09-1183-s001.zip › FinalCode/Dataset/DB4_B/110_2.tif]

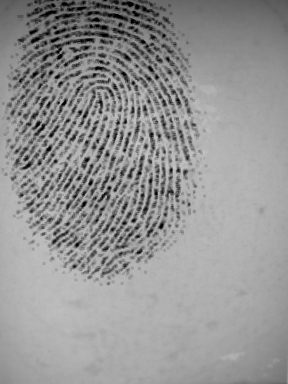

Supplement: Supplemental Information 1 [file peerj-cs-09-1183-s001.zip › FinalCode/Dataset/DB4_B/109_3.tif]

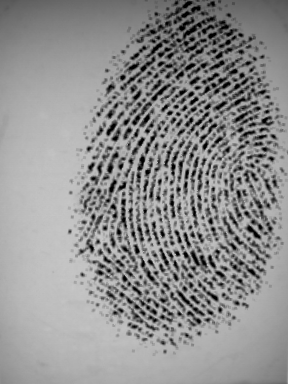

Supplement: Supplemental Information 1 [file peerj-cs-09-1183-s001.zip › FinalCode/Dataset/DB4_B/108_4.tif]

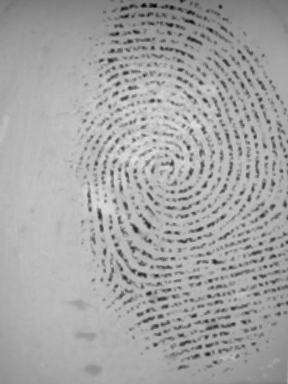

Supplement: Supplemental Information 1 [file peerj-cs-09-1183-s001.zip › FinalCode/Dataset/DB4_B/104_8.tif]

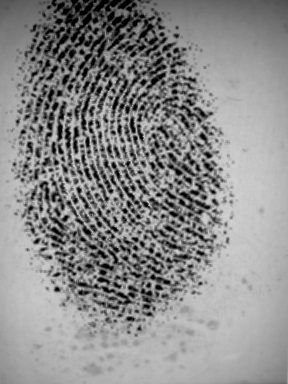

Supplement: Supplemental Information 1 [file peerj-cs-09-1183-s001.zip › FinalCode/Dataset/DB4_B/108_5.tif]

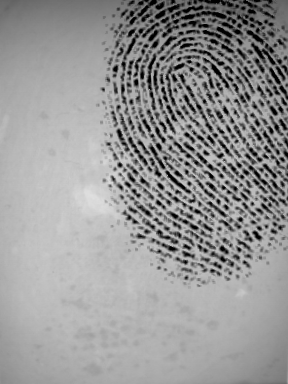

Supplement: Supplemental Information 1 [file peerj-cs-09-1183-s001.zip › FinalCode/Dataset/DB4_B/108_7.tif]

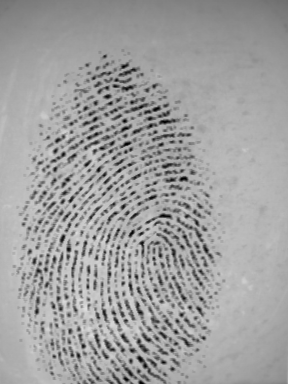

Supplement: Supplemental Information 1 [file peerj-cs-09-1183-s001.zip › FinalCode/Dataset/DB4_B/106_8.tif]

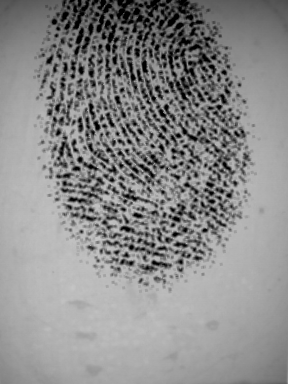

Supplement: Supplemental Information 1 [file peerj-cs-09-1183-s001.zip › FinalCode/Dataset/DB4_B/108_6.tif]

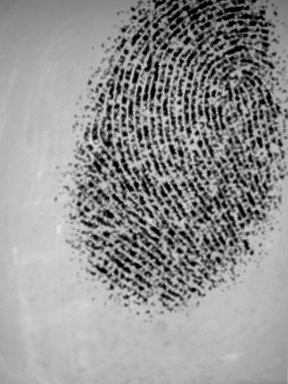

Supplement: Supplemental Information 1 [file peerj-cs-09-1183-s001.zip › FinalCode/Dataset/DB4_B/108_2.tif]

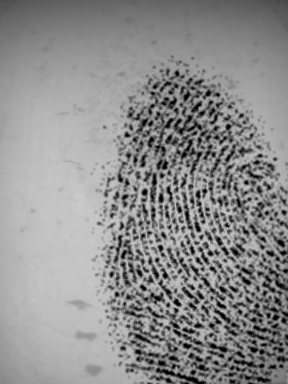

Supplement: Supplemental Information 1 [file peerj-cs-09-1183-s001.zip › FinalCode/Dataset/DB4_B/108_3.tif]

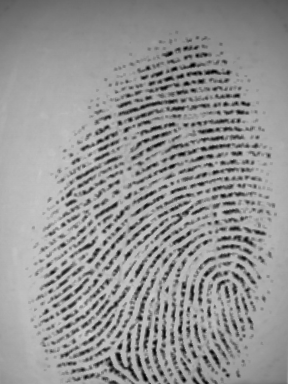

Supplement: Supplemental Information 1 [file peerj-cs-09-1183-s001.zip › FinalCode/Dataset/DB4_B/102_8.tif]

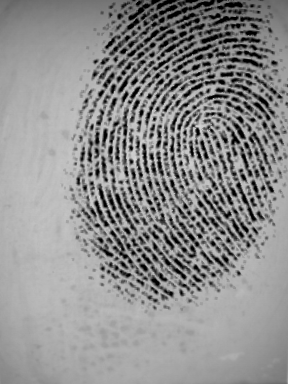

Supplement: Supplemental Information 1 [file peerj-cs-09-1183-s001.zip › FinalCode/Dataset/DB4_B/108_1.tif]

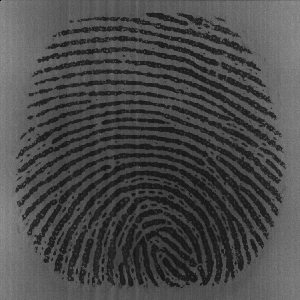

Supplement: Supplemental Information 1 [file peerj-cs-09-1183-s001.zip › FinalCode/Dataset/DB3_B/102_6.tif]

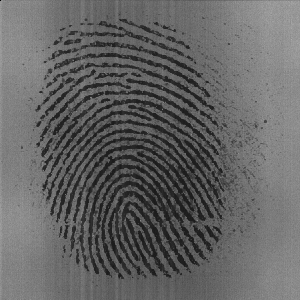

Supplement: Supplemental Information 1 [file peerj-cs-09-1183-s001.zip › FinalCode/Dataset/DB3_B/106_2.tif]

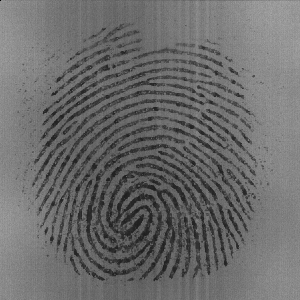

Supplement: Supplemental Information 1 [file peerj-cs-09-1183-s001.zip › FinalCode/Dataset/DB3_B/104_1.tif]

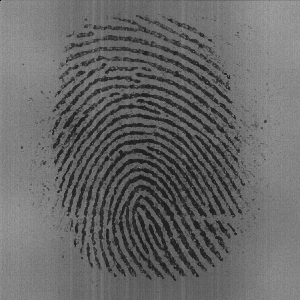

Supplement: Supplemental Information 1 [file peerj-cs-09-1183-s001.zip › FinalCode/Dataset/DB3_B/106_3.tif]

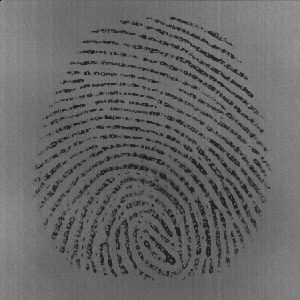

Supplement: Supplemental Information 1 [file peerj-cs-09-1183-s001.zip › FinalCode/Dataset/DB3_B/102_7.tif]

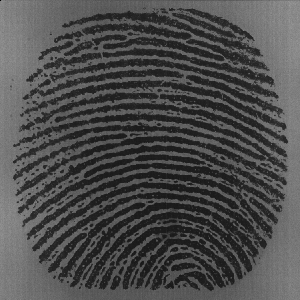

Supplement: Supplemental Information 1 [file peerj-cs-09-1183-s001.zip › FinalCode/Dataset/DB3_B/102_5.tif]

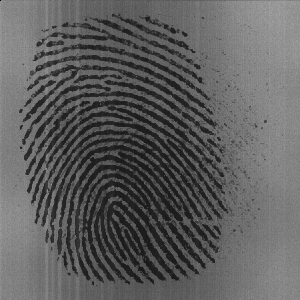

Supplement: Supplemental Information 1 [file peerj-cs-09-1183-s001.zip › FinalCode/Dataset/DB3_B/106_1.tif]

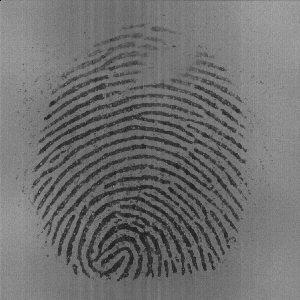

Supplement: Supplemental Information 1 [file peerj-cs-09-1183-s001.zip › FinalCode/Dataset/DB3_B/104_3.tif]

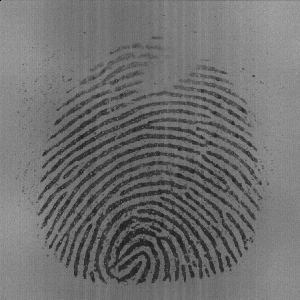

Supplement: Supplemental Information 1 [file peerj-cs-09-1183-s001.zip › FinalCode/Dataset/DB3_B/104_2.tif]

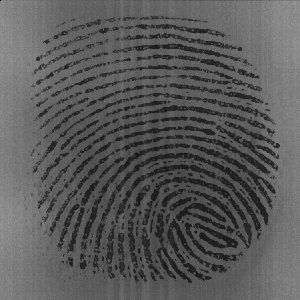

Supplement: Supplemental Information 1 [file peerj-cs-09-1183-s001.zip › FinalCode/Dataset/DB3_B/102_4.tif]

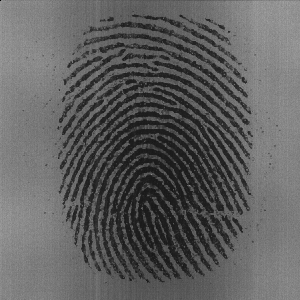

Supplement: Supplemental Information 1 [file peerj-cs-09-1183-s001.zip › FinalCode/Dataset/DB3_B/106_4.tif]

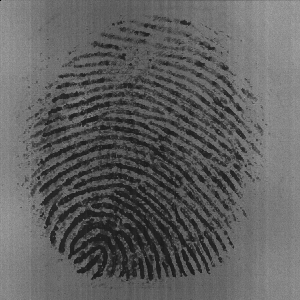

Supplement: Supplemental Information 1 [file peerj-cs-09-1183-s001.zip › FinalCode/Dataset/DB3_B/104_6.tif]

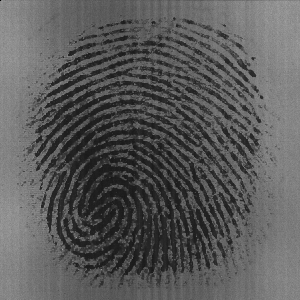

Supplement: Supplemental Information 1 [file peerj-cs-09-1183-s001.zip › FinalCode/Dataset/DB3_B/104_7.tif]

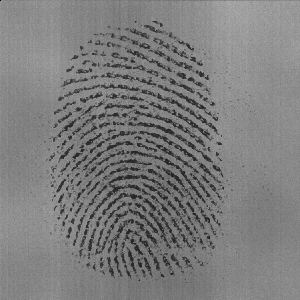

Supplement: Supplemental Information 1 [file peerj-cs-09-1183-s001.zip › FinalCode/Dataset/DB3_B/106_5.tif]

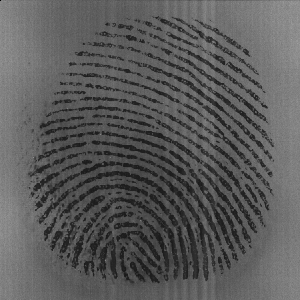

Supplement: Supplemental Information 1 [file peerj-cs-09-1183-s001.zip › FinalCode/Dataset/DB3_B/102_1.tif]

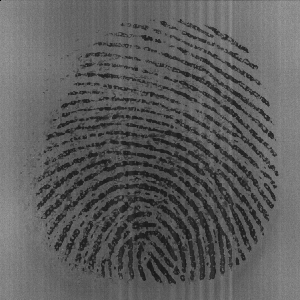

Supplement: Supplemental Information 1 [file peerj-cs-09-1183-s001.zip › FinalCode/Dataset/DB3_B/102_3.tif]

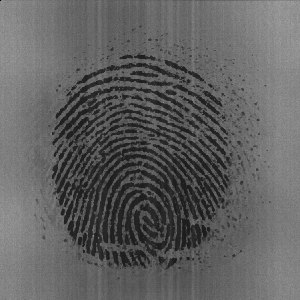

Supplement: Supplemental Information 1 [file peerj-cs-09-1183-s001.zip › FinalCode/Dataset/DB3_B/108_8.tif]

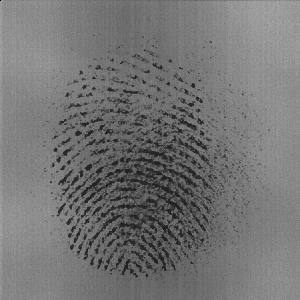

Supplement: Supplemental Information 1 [file peerj-cs-09-1183-s001.zip › FinalCode/Dataset/DB3_B/106_7.tif]

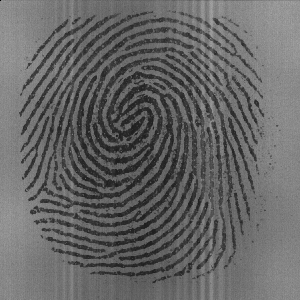

Supplement: Supplemental Information 1 [file peerj-cs-09-1183-s001.zip › FinalCode/Dataset/DB3_B/104_5.tif]

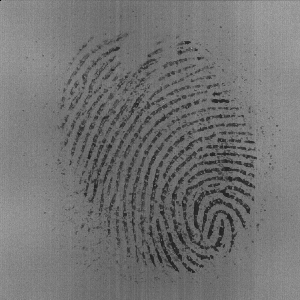

Supplement: Supplemental Information 1 [file peerj-cs-09-1183-s001.zip › FinalCode/Dataset/DB3_B/104_4.tif]
